# Supplementary figures and images for: Many but not all deep neural network audio models capture brain responses and exhibit correspondence between model stages and brain regions
Source: PLoS Biol. 2023 Dec 13;21(12):e3002366. doi: 10.1371/journal.pbio.3002366 (PMC10718467; doi:10.1371/journal.pbio.3002366)

**A** NH2015 fMRI RDMs

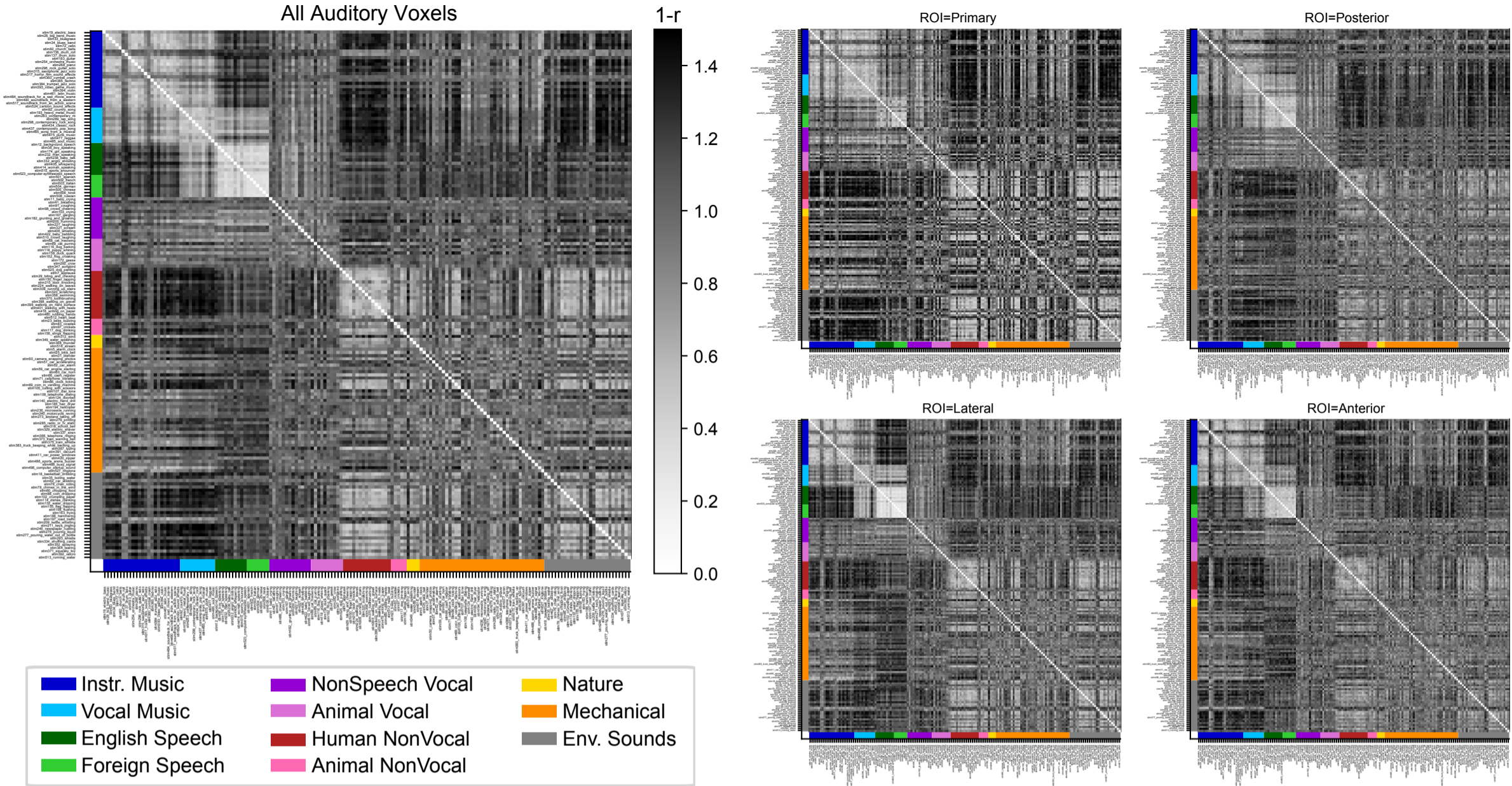

**B** B2021 fMRI RDMs

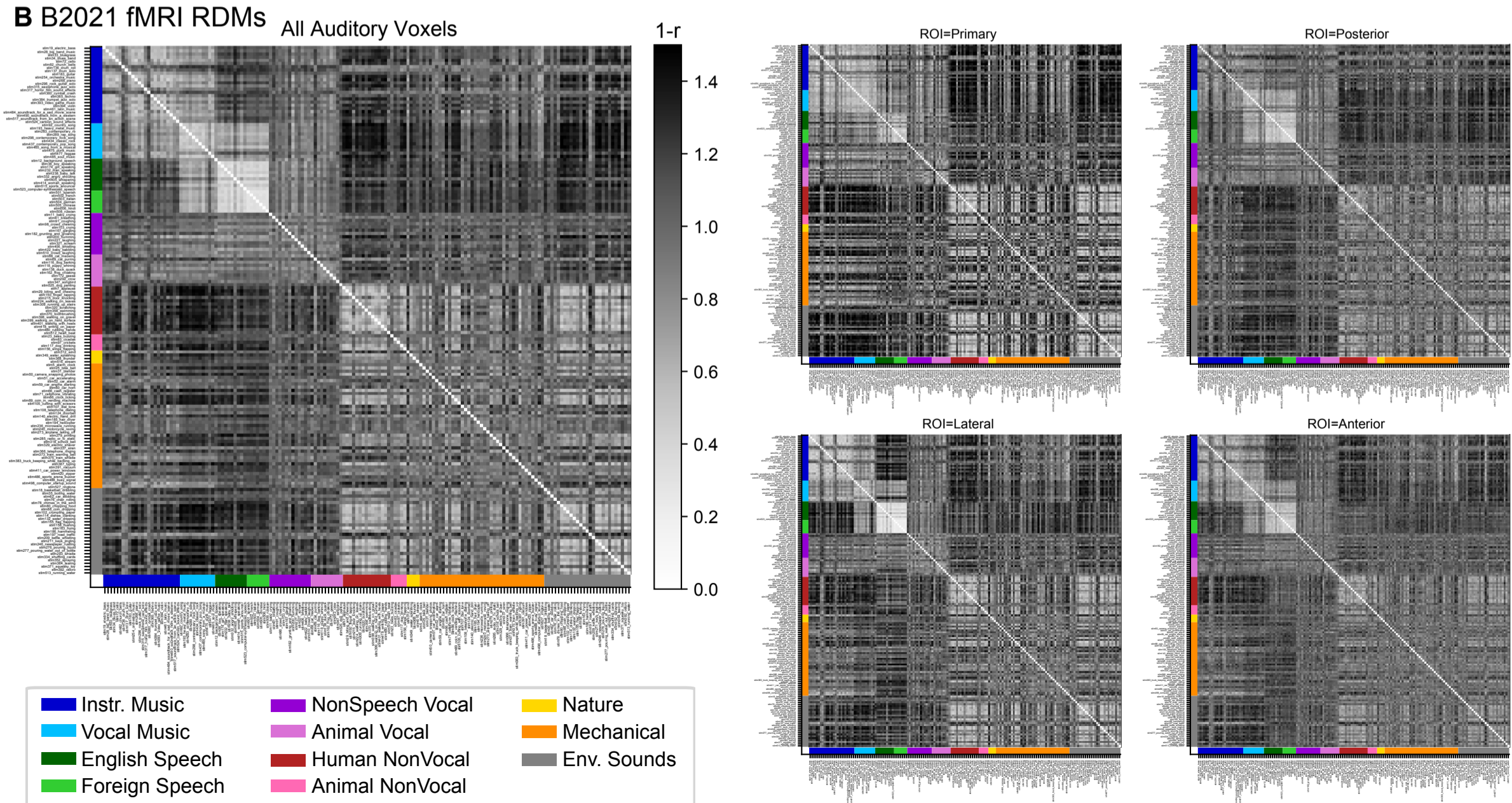

Supplement: S1 Fig — For visualization purposes, the RDMs are computed as 1 minus the Pearson correlation coefficient between the 3-scan average BOLD responses for pairs of sounds. RDMs are computed for all sound-responsive voxels (left) and using only a subset of voxels for each of the anatomical ROIs (right). Sounds are grouped by sound categories (included in colors on the axis). Data and code with which to reproduce results are available at https://github.com/gretatuckute/auditory_brain_dnn. (PDF) [file pbio.3002366.s001.pdf]

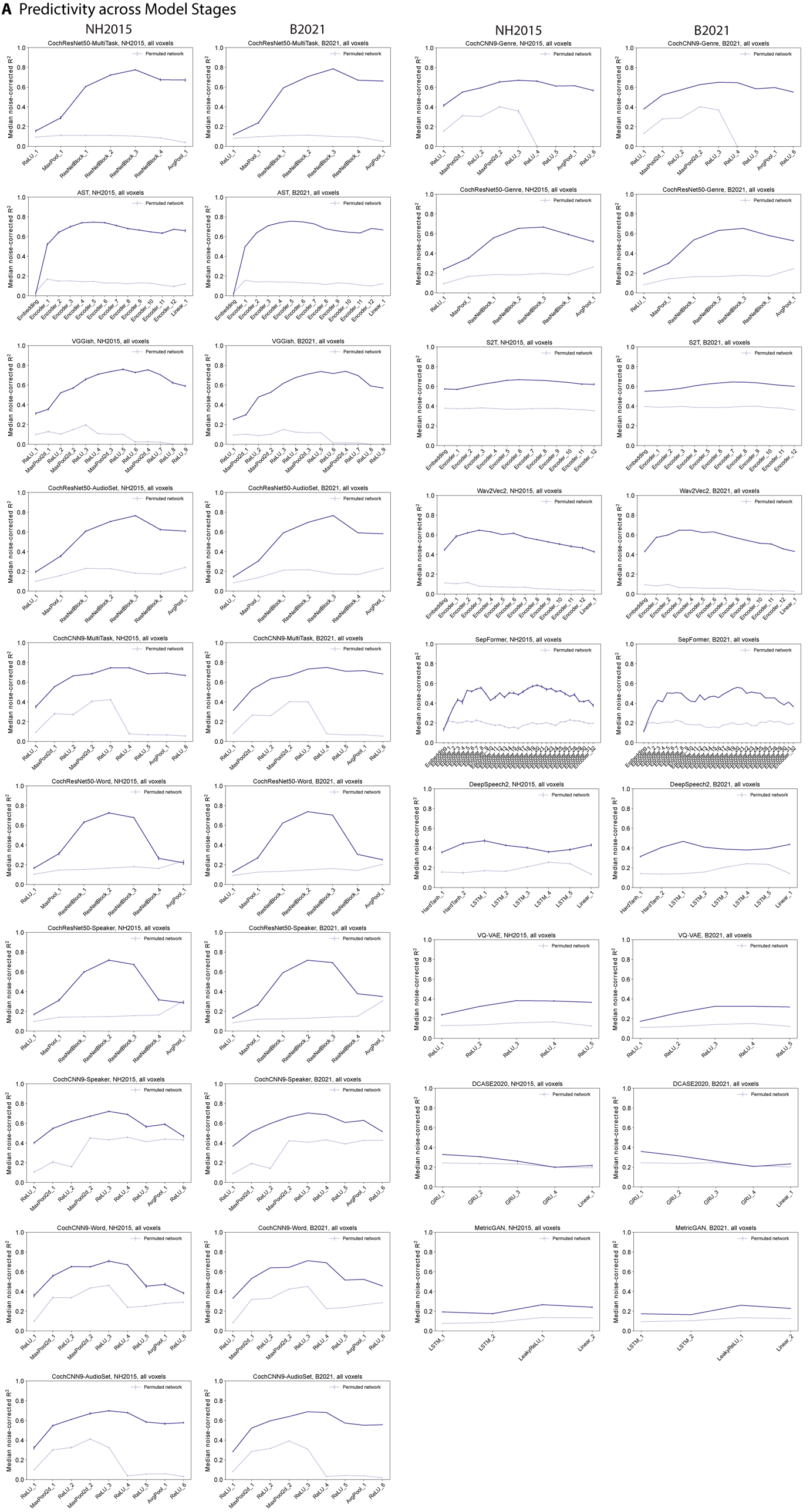

Supplement: S2 Fig — Explained variance was measured for each voxel, and the aggregated median variance explained across all voxels in auditory cortex was obtained. This aggregated median variance explained is plotted for all candidate models (n = 19) for both fMRI datasets. The model plots are sorted according to overall model performance (median noise-corrected R2 for NH2015 in Fig 2A in the main text), meaning that the first subplot shows the best-performing model, CochResNet50-MultiTask, and the last subplot shows the worst-performing model, MetricGAN. Dark lines show the trained networks, and lighter lines show the control networks with permuted weights. Error bars are within-participant SEM. Error bars are smaller for the B2021 dataset because of the larger number of participants (n = 20 vs. n = 8). We note that some of the variation in predictivity across model stages in the models with permuted weights could be driven by the receptive field sizes at different stages, which are partly a function of the model architecture. Data and code with which to reproduce results are available at https://github.com/gretatuckute/auditory_brain_dnn. (TIF) [file pbio.3002366.s002.tif]

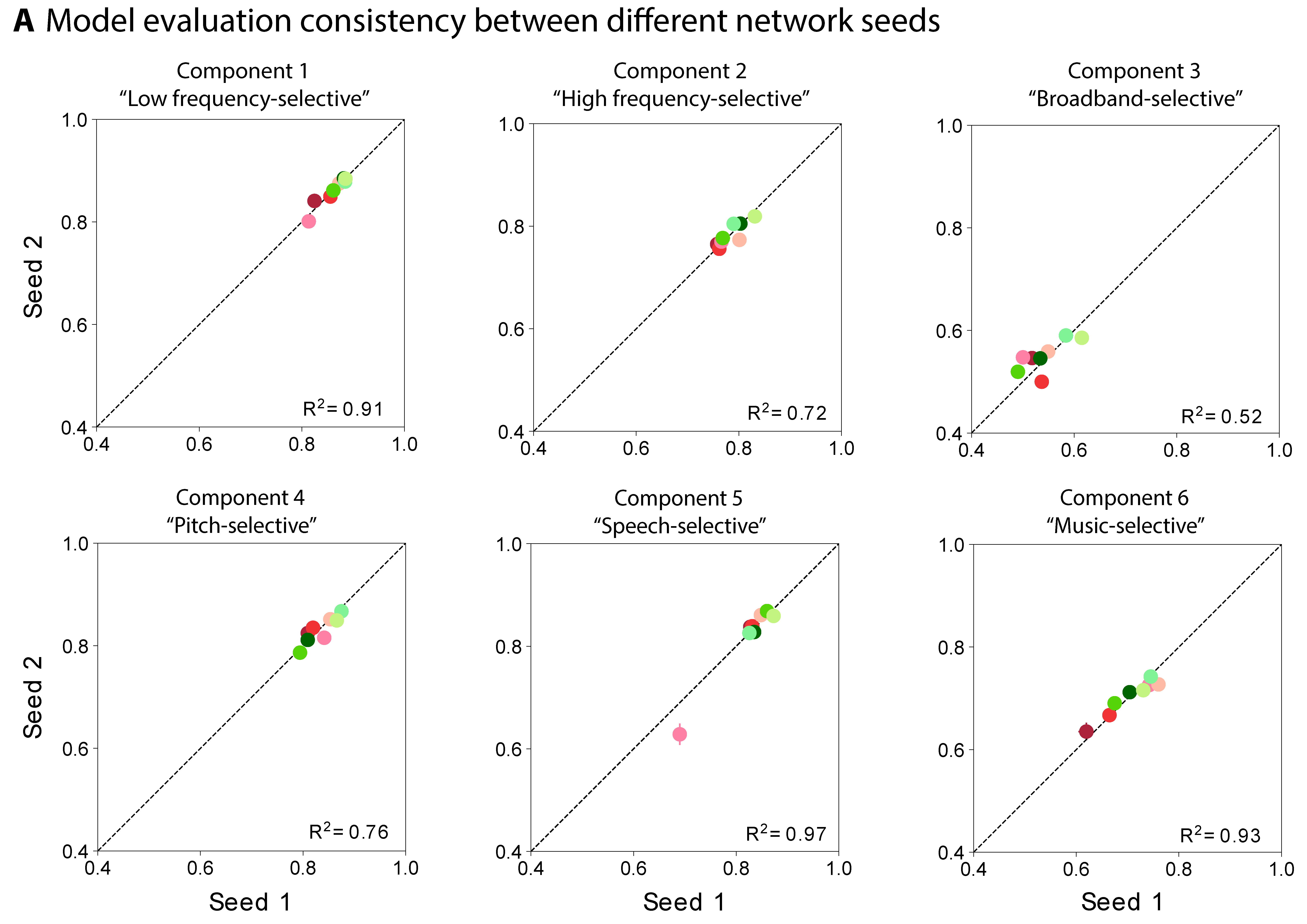

Supplement: S3 Fig — We trained the in-house models from 2 different random seeds. The variance explained for the first seed is plotted on the x-axis and for the second seed on the y-axis. Each data point represents a model using with the same color correspondence as in Fig 2 in the main text. Variance explained was obtained from the best-predicting stage of each model for each component, selected using independent data. Error bars are SEM over iterations of the model stage selection procedure (see Methods; Component modeling). Data and code with which to reproduce results are available at https://github.com/gretatuckute/auditory_brain_dnn. (TIF) [file pbio.3002366.s003.tif]

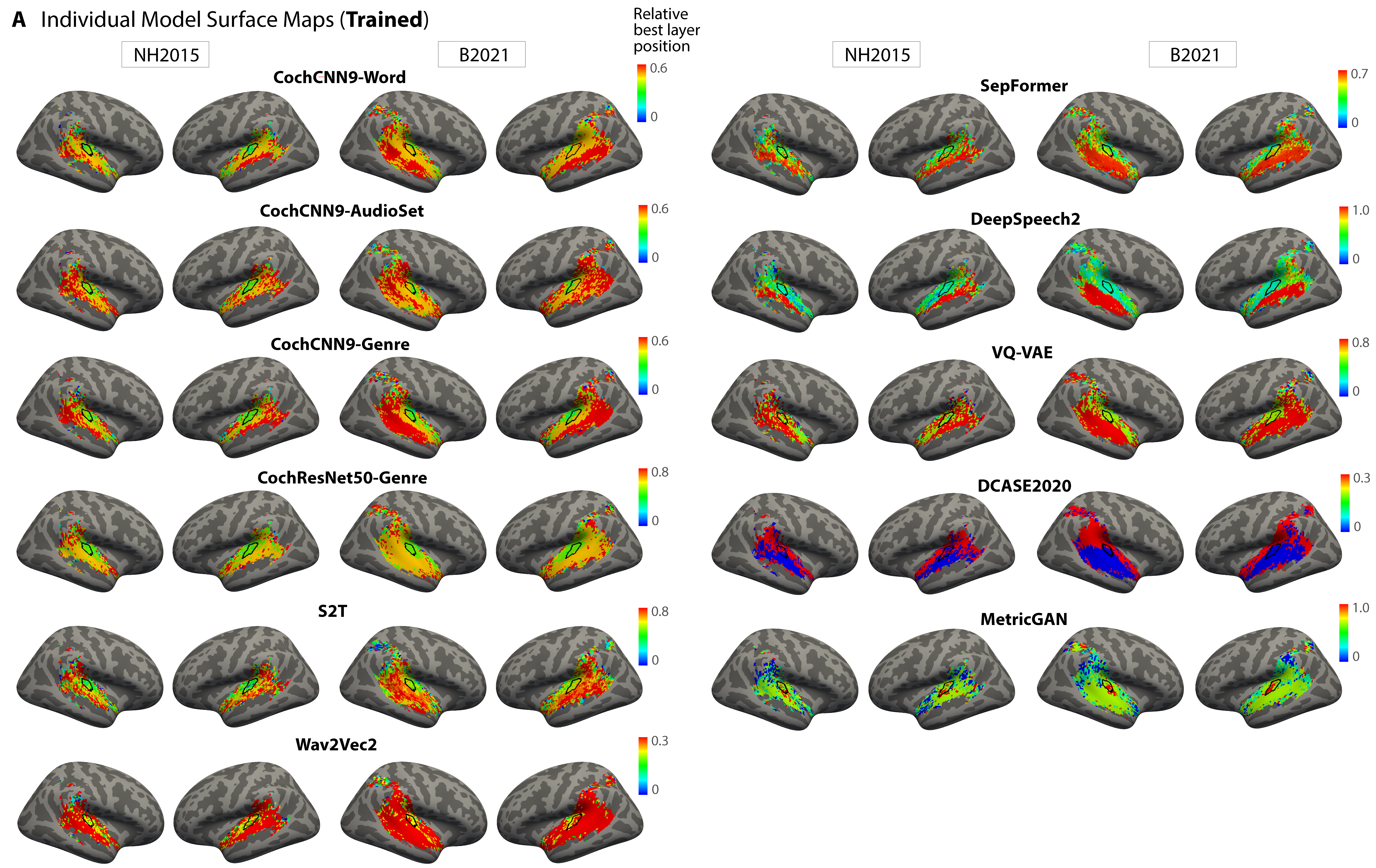

Supplement: S4 Fig — The figure shows surface maps for trained models that are not included in Fig 6A in the main text (which featured the n = 8 best-predicting models, leaving the n = 11 models shown here). The plots are sorted according to overall model predictivity (the quantity plotted in Fig 2A in the main text). As in Fig 6A in the main text, the plots show the model stage that best predicts each voxel as a surface map (FsAverage) (median best stage across participants). We assigned each model stage a position index between 0 and 1. The color scale limits were set to extend from 0 to the stage beyond the most common best stage (across voxels). Data and code with which to reproduce results are available at https://github.com/gretatuckute/auditory_brain_dnn. (TIF) [file pbio.3002366.s004.tif]

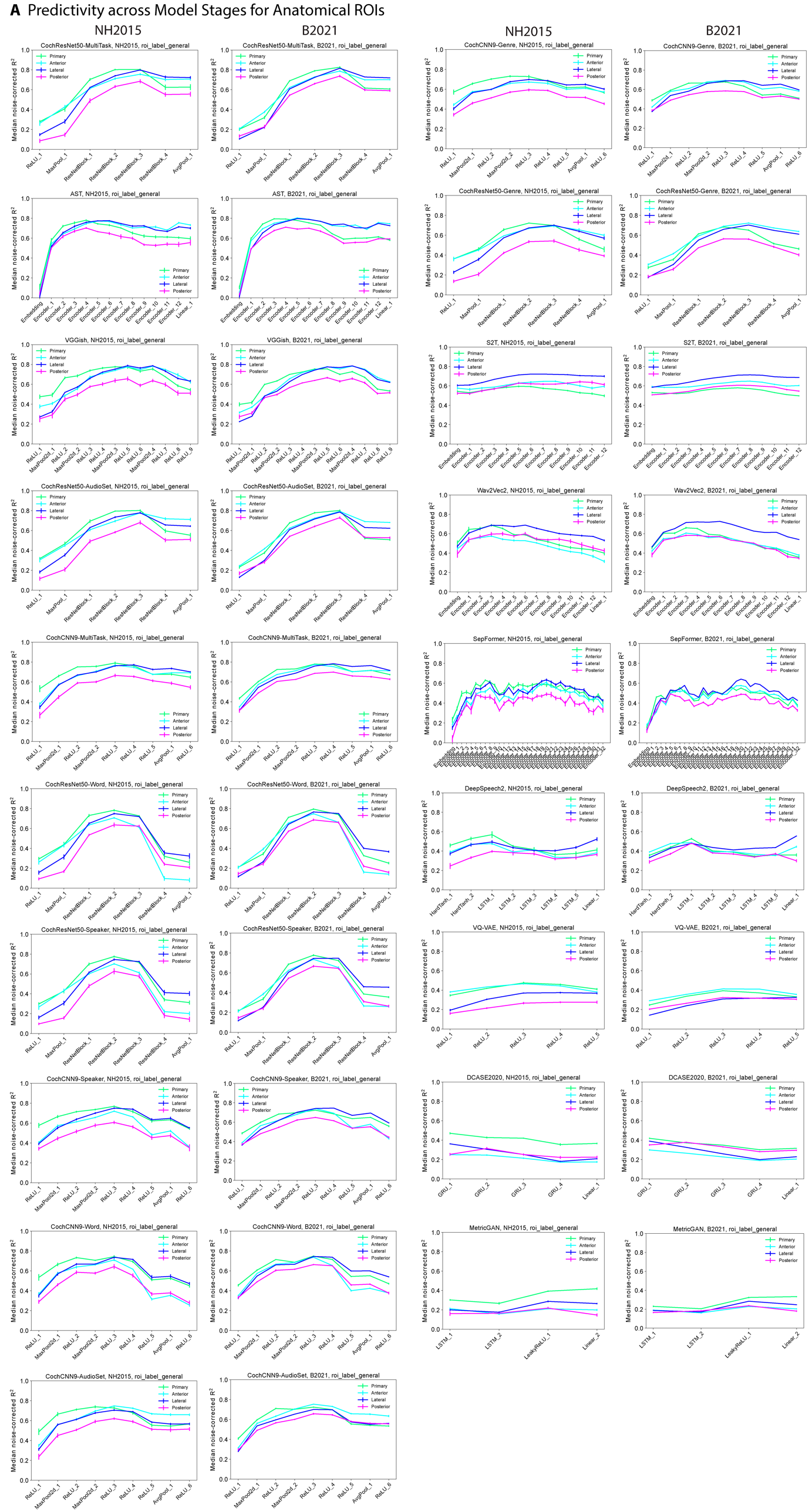

Supplement: S6 Fig — Explained variance was measured for each voxel, and the aggregated median variance explained across each of the 4 anatomical ROIs (primary, anterior, lateral, posterior) was obtained. This aggregated median variance explained is plotted for all stages of all candidate models (n = 19) for both fMRI datasets. The model plots are sorted according to overall model predictivity (median noise-corrected R2 for NH2015 in Fig 2A in the main text; same model order as in S2 Fig). Error bars are within-participant SEM. Error bars are smaller for the B2021 dataset because of the larger number of participants (20 vs. 8). Data and code with which to reproduce results are available at https://github.com/gretatuckute/auditory_brain_dnn. (TIF) [file pbio.3002366.s006.tif]

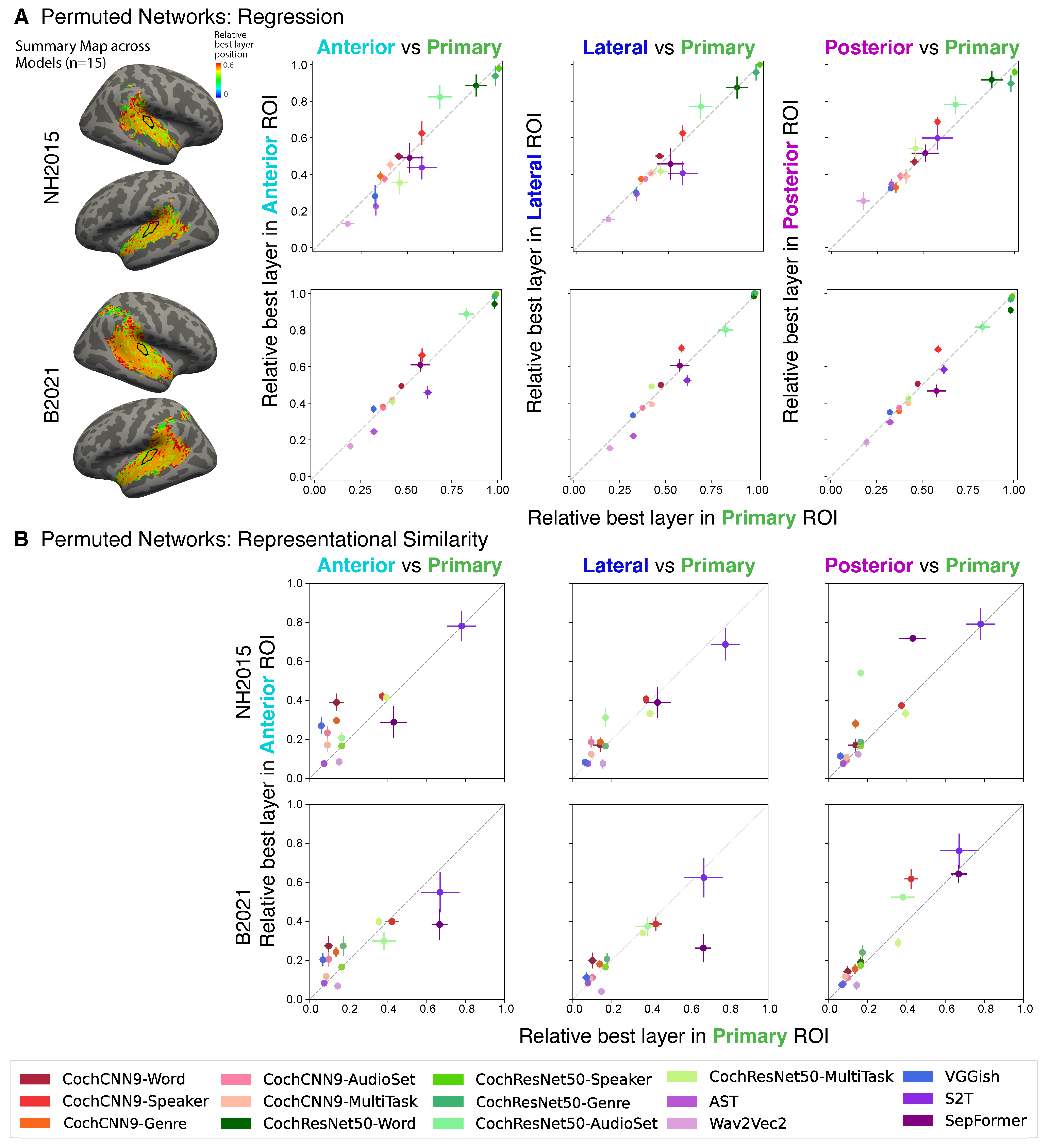

Supplement: S7 Fig — This figure mirrors Fig 7 in the main text, which shows the quantification of model-stage-region correspondence across trained models. (A) As in Fig 7 in the main text, we obtained the median best-predicting stage for each model within 4 anatomical ROIs (illustrated in Fig 7A, main text): primary auditory cortex (x-axis in each plot in panels A and B) and anterior, lateral, and posterior non-primary regions (y-axes in panels A and B). We performed the analysis on each of the 2 fMRI data sets, including each model that outpredicted the baseline model in Fig 2A in the main text (n = 15 models). Each data point corresponds to a model with permuted weights, with the same color correspondence as in Fig 2 in the main text. None of the 6 possible comparisons (2 datasets × 3 non-primary ROIs) were statistically significant even without correction for multiple comparisons, p > 0.16 in all cases (Wilcoxon signed rank tests, two-tailed). (B) Same analysis as panel A but with the best-matching model stage determined by correlations between the model and ROI representational dissimilarity matrices. None of the 6 possible comparisons were statistically significant even without correction for multiple comparisons, p > 0.07 in all cases (Wilcoxon signed rank tests, two-tailed). Data and code with which to reproduce results are available at https://github.com/gretatuckute/auditory_brain_dnn. (TIF) [file pbio.3002366.s007.tif]

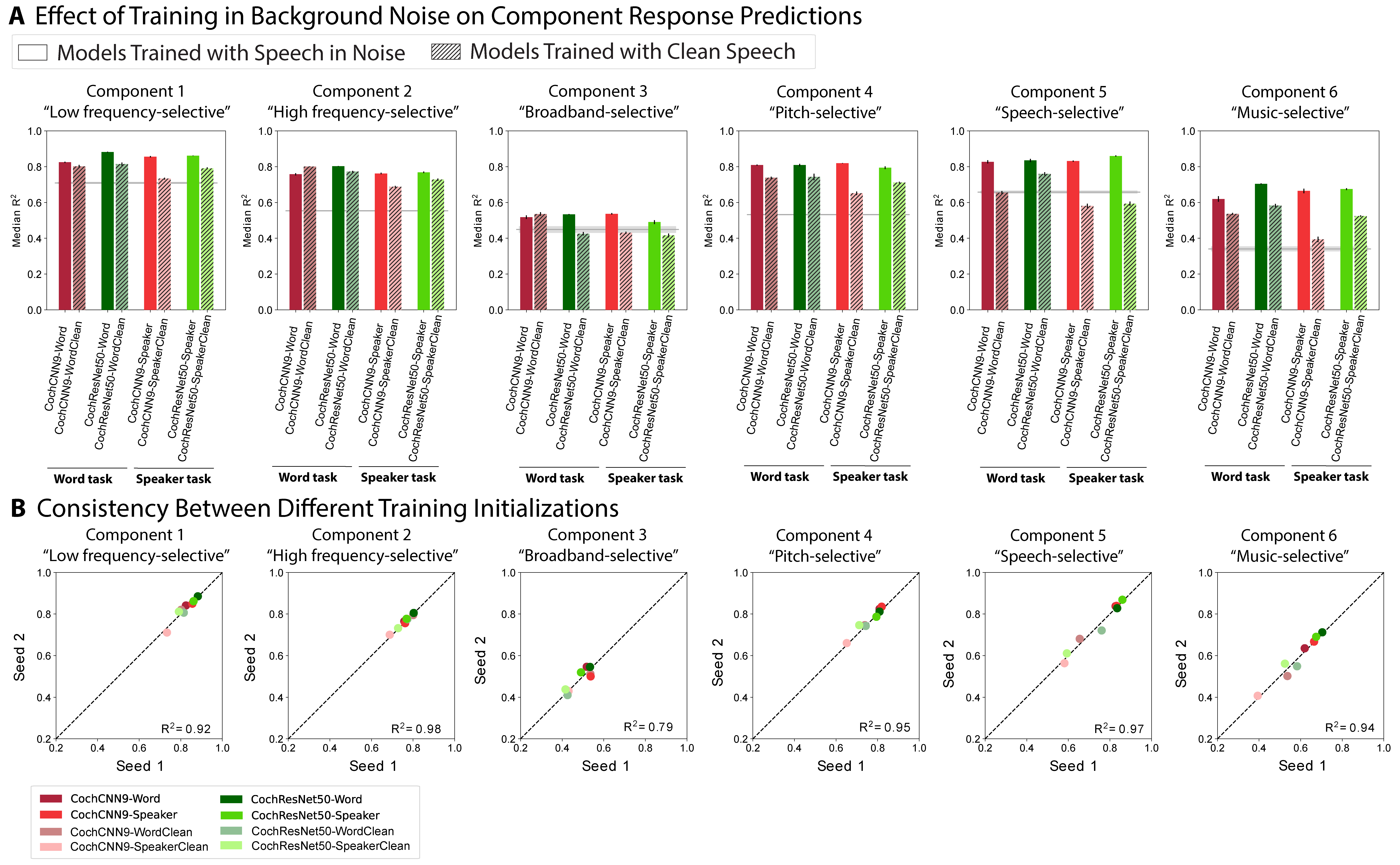

Supplement: S8 Fig — (A) Variance explained was obtained from the best-predicting stage of each model for each component, selected using independent data. Models trained in the presence of background noise are shown in the same color scheme as in Fig 2 in the main text; models trained with clean speech are shown with hashing. Grey line shows variance explained by the SpectroTemporal baseline model. Error bars are SEM over iterations of the model stage selection procedure (see Methods; Component modeling). (B) We trained the models from 2 different random seeds. The variance explained for the first seed is plotted on the x-axis and for the second seed on the y-axis. Each data point represents a model. Data and code with which to reproduce results are available at https://github.com/gretatuckute/auditory_brain_dnn. (TIF) [file pbio.3002366.s008.tif]

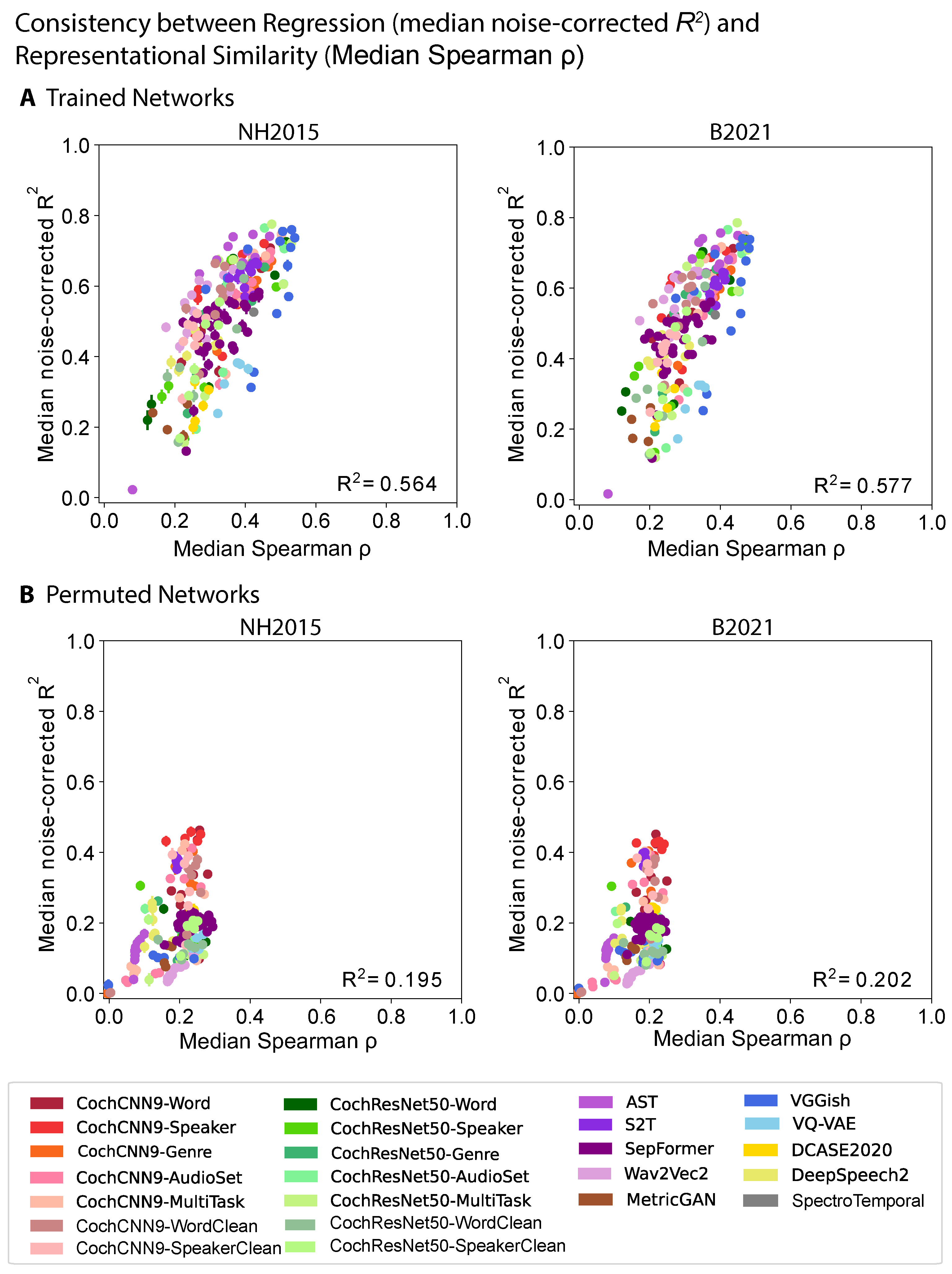

Supplement: S10 Fig — (A) Correlation between the regression-based metric (median noise-corrected R2) and the representational similarity metric (median Spearman correlation) across trained network stages for the NH2015 and B2021 datasets. Each data point corresponds to a network stage, with the same color correspondence as in Fig 2 in the main text. (B) Same as panel A, but for permuted network stages. All unique models in the study were included in this analysis (n = 20 models in Fig 2 in the main text plus n = 4 models trained on the word and speaker tasks without background noise from Fig 8 in the main text, i.e., n = 24 models in total). Data and code with which to reproduce results are available at https://github.com/gretatuckute/auditory_brain_dnn. (TIF) [file pbio.3002366.s010.tif]
